# P02.02. Efficacy of a topical 0.1% Capsaicin hydrogel patch to treat chronic neck pain: a double-blind randomized clinical trial

**DOI:** 10.1186/1472-6882-12-S1-P58

**Published:** 2012-06-12

**Authors:** M Brodsky, J Cho, J Fang, E Kim, Y Cho, M Song

**Affiliations:** 1 Stamford Hospital, Columbia University, Stamford, USA; 2 Kyung Hee University, Oriental Rehabilitation Medicine, Seoul, Republic of Korea; 3 Chang Gung University, Pharmaceutics Laboratory, Teipai, Taiwan

## Purpose

Myofascial trigger points are a common component of musculoskeletal neck pain. Capsaicin, a vanilloid 1 (TRPV1) receptor agonist, has been shown to induce pain in human tendon tissues and to increase trigger point sensitivity in humans. TRPV1 receptor agonists, with repeated use, inhibit the initiation of transmission of pain-related neurotransmitters and may alleviate pain. This study evaluated the efficacy of a hydrogel patch containing capsaicin 0.1% compared to a placebo hydrogel patch without capsaicin to treat chronic myofascial neck pain.

## Methods

Sixty-one subjects between the ages of 18 and 65 years old with at least 3 months of myofascial neck pain were recruited. Participants were randomized to apply either capsaicin 0.1% (500mcg) hydrogel patches or hydrogel control patches without capsaicin to each side of the neck and shoulder girdle area for 12 hours each day for a 4-week treatment period. The following instruments were administered at baseline, at 2-weeks after the start of treatment, at the conclusion of the 4-week treatment, and 4 weeks after the treatment period: (1) change on a visual analogue scale (VAS), the primary outcome measure; (2) Neck Disability Index (NDI); (3) Beck’s Depression inventory (BDI); (4) Short Form 36 (SF-36) Korean version; and (5) Euroqol 5-D(EQ-5D) Health Questionnaire.

## Results

Fifty-seven subjects completed the study. The mean VAS scores were significantly decreased at 2, 4 and 8 weeks after the start of the intervention in both groups. However, there was no significant difference in VAS score between the two groups or in any of the other outcome measures.

## Conclusion

Given the finding that subjects had a statistically significant improvement with the 0.1% capsaicin hydrogel patch compared to baseline, further studies may be warranted. Future investigation may assess capsaicin as an adjunct to the treatment of myofascial neck pain with other self-care, medication, and office based approaches.

